# Tetra­hydro­alstonine

**DOI:** 10.1107/S1600536813021168

**Published:** 2013-08-07

**Authors:** Xavier Cachet, François-Hugues Porée, Sylvie Michel, Pascale Lemoine

**Affiliations:** a Laboratoire de Pharmacognosie, UMR CNRS 8638, Faculté des Sciences Pharmaceutiques et Biologiques de Paris Descartes, 4, avenue de l’Observatoire, 75270 Paris Cedex 06, France; b Laboratoire de Cristallographie et RMN biologiques, UMR CNRS 8015, Faculté des Sciences Pharmaceutiques et Biologiques de Paris Descartes, 4, avenue de l’Observatoire, 75270 Paris Cedex 06, France

## Abstract

In the title compound, C_21_H_24_N_2_O_3_ [systematic name: methyl (20α)-16,17-dide­hydro-19α-methyl-18-oxayohimban-16-carb­oxy­l­ate], the mol­ecule adopts an L-type conformation. The crystal packing is governed by one N—H⋯π and one C—H⋯π inter­actions. The crystal cohesion is ensured by inter­molecular van der Waals contacts [shortest O⋯O contact = 3.199 (2) Å].

## Related literature

For the extraction of tetra­hydro­alstonine (THA) from natural sources, see: Zenk & Juenger (2007 ▶); Mandal *et al.* (1983 ▶); Langlois *et al.* (1979 ▶). For stereochemistry studies, see: Wenkert *et al.* (1961 ▶); Wenkert & Roychaudhuri (1957 ▶); Shamma & Richey (1963 ▶); Lounasmaa & Kan (1980 ▶); Höfle *et al.* (1980 ▶). For the semisynthesis, see: Poirot (2007 ▶); Beziat & Hatinguais (1977 ▶); Zsadon *et al.* (1979 ▶); Guéritte *et al.* (1983 ▶); Hemscheidt & Zenk (1985 ▶) and for synthetic studies, see: Gutzwiller *et al.* (1971 ▶); Wenkert *et al.* (1976 ▶); Zou *et al.* (2010 ▶). For the biological activity of TMA, see Zou *et al.* (2010 ▶); Sharma *et al.* (1988 ▶). For a related structure, see: Laus & Wurst (2008 ▶).
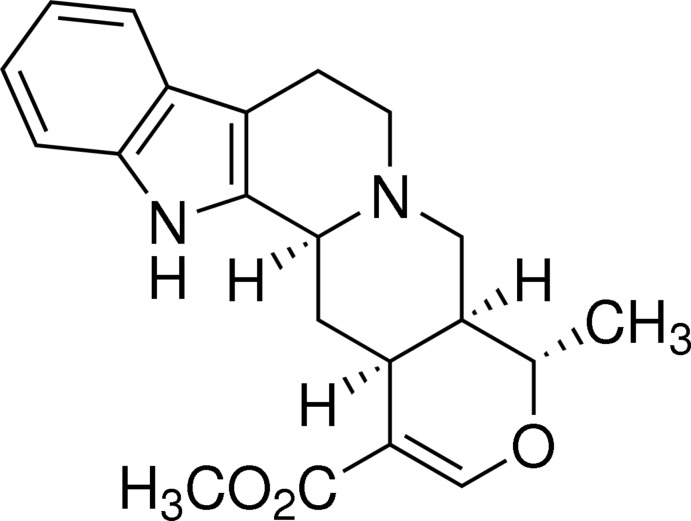


## Experimental

### 

#### Crystal data

C_21_H_24_N_2_O_3_
*M* *_r_* = 352.42
Orthorhombic, 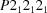

*a* = 6.719 (1) Å
*b* = 8.169 (2) Å
*c* = 34.120 (5) Å
*V* = 1872.8 (6) Å^3^
*Z* = 4
Mo *K*α radiation
μ = 0.08 mm^−1^
*T* = 293 K
0.50 × 0.30 × 0.10 mm

#### Data collection

Nonius KappaCCD diffractometer
Absorption correction: multi-scan (*COLLECT*; Nonius, 2004 ▶) *T*_min_ = 0.982, *T*_max_ = 0.992
3300 measured reflections
3300 independent reflections
2537 reflections with *I* > 2σ(*I*)

#### Refinement

*R*[*F* ^2^ > 2σ(*F* ^2^)] = 0.044
*wR*(*F* ^2^) = 0.114
*S* = 1.01
3300 reflections
247 parameters
H atoms treated by a mixture of independent and constrained refinement
Δρ_max_ = 0.11 e Å^−3^
Δρ_min_ = −0.12 e Å^−3^
Absolute structure parameter: −0.3 (17)

### 

Data collection: *COLLECT* (Nonius, 2004 ▶); cell refinement: *SCALEPACK* (Otwinowski & Minor, 1997 ▶); data reduction: *DENZO* (Otwinowski & Minor, 1997 ▶) and *SCALEPACK*; program(s) used to solve structure: *SIR92* (Altomare *et al.*, 1994 ▶); program(s) used to refine structure: *SHELXL97* (Sheldrick, 2008 ▶); molecular graphics: *ORTEP-3 for Windows* (Farrugia, 2012 ▶); software used to prepare material for publication: *WinGX* (Farrugia, 2012 ▶).

## Supplementary Material

Crystal structure: contains datablock(s) I. DOI: 10.1107/S1600536813021168/bq2388sup1.cif

Structure factors: contains datablock(s) I. DOI: 10.1107/S1600536813021168/bq2388Isup2.hkl

Additional supplementary materials:  crystallographic information; 3D view; checkCIF report

## Figures and Tables

**Table 1:** Hydrogen-bond geometry (Å, °) *Cg*1 and *Cg*2 are the centroids of the C8–C13 and C2/C7/C8/C13/N1 rings, respectively.

*D*—H⋯*A*	*D*—H	H⋯*A*	*D*⋯*A*	*D*—H⋯*A*
N1—H1⋯*Cg*1^i^	0.86	2.85	3.550 (2)	139
C6—H6*A*⋯*Cg*2^ii^	0.97	2.8	3.429 (3)	121

Symmetry codes: (i) 
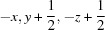
; (ii) 
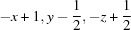
.

**Fig. 1.**
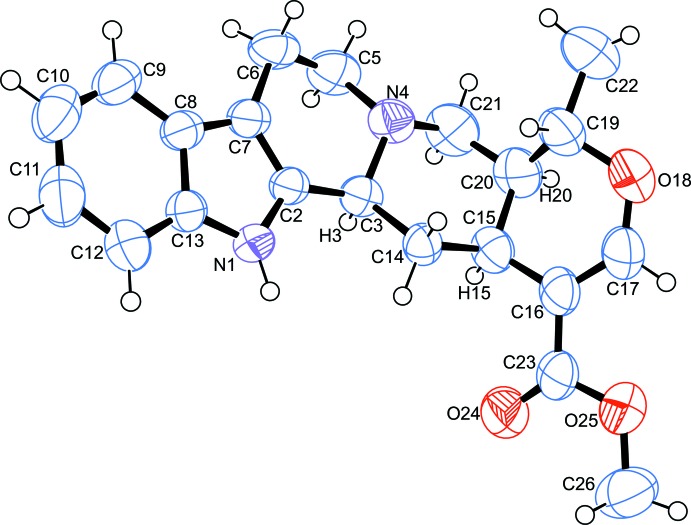
Molecular view of the compound showing atomic numbering. Displacements ellipsoids at the 50% probability level.

## supplementary crystallographic information

### 1. Comment

Tetrahydroalstonine (THA) is an indolomonoterpenoid alkaloid possessing a 5-fused ring corynanthean type skeleton (Höfle *et al.* 1980; Zenk & Juenger 2007). Depending on the stereochemistry at the positions C3, C15, C19 and C20, several subgroups of corynanthe alkaloids have been distinguished (Lounasmaa & Kan, 1980). THA belongs to the alloheteroyohimbine subgroup together with rauniticine (Shamma & Richey 1963). Its distribution is restricted to plants belonging to the sole plants of the Apocynaceae, Rubiaceae and Loganiaceae families. THA constitutes the main by-product of the marketed drug raubasine (ajmalicine) industrial production (Poirot 2007; Beziat & Hatinguais 1977; Zsadon *et al.* 1979). Indeed, this molecule is readily available in a large amount by the reduction with NaBH_4_ of serpentine present in the underground parts of *Catharanthus roseus* (Mandal *et al.* 1983). This reaction directly carried out on a total alkaloid extract also affords THA resulting from alstonine reduction. Furthermore, this method followed by the isolation of THA and raubasine has been employed for a convenient determination of the alstonine and serpentine respective content in the aerial parts of *Catharanthus ovalis* (Langlois *et al.* 1979). The structure of THA has been elucidated after extensive NMR studies during the period 1950–1960 (Wenkert & Roychaudhuri 1957; Wenkert *et al.* 1961). In the corynanthe series, only the crystal structure of akuammigine (as picrate hydrate) has been previously reported (Laus & Wurst 2008). There have been only a few reports of the THA biological activities (Zou *et al.* 2010; Sharma *et al.* 1988). The X-ray data of THA confirmed the relative *allo* configuration of the C3, C15, C19 and C20 stereocenters. With reference of the known (15*S*)- configuration due to the biosynthesis, the absolute configuration of the other stereocenters is thus given by (3*S*, 19S, 20S). The aromatic rings A (C8-C13) and B (C2/C7/C8/C13/N1) are planar. Ring C (C2/C3/N4/C5-C7) adopts a half-chair conformation with N4 above the medium A/B plane by 0.437 (4) Å and C5 below the medium A/B plane by 0.334 (4) Å, respectively. The C/D ring is a *trans*-fused quinolizidine. Ring D (C3/C14/C15/C20/C21/N4) is a regular chair with H—C3 α axial. The D/E rings are *cis*-fused; H—C15 and H—C20 are α, the first one being in axial position and the second in equatorial. Ring E (C15-C20) is a half-chair with C19 above the median plan (C15—C16—C17—O18) by -0.273 (5) Å and C20 below the same plan by 0.497 (4) Å. The α Me—C19 group adopts a pseudoequatorial position (Figure 1.). The crystal packing of the compound is governed by one N—H···*Cg* (π ring, *Cg* centroid of ring A) interaction and one C—H···*Cg* (π ring, *Cg* centroid of ring B) interaction. The crystal cohesion is ensured by intermolecular van der Waals contacts, the shortest is equal to 3.199 (2) Å for O18 and O24.

### 2. Experimental

The typical conditions used for the semi-synthesis were followed. The pulverized dried roots of *Catharanthus roseus* (20.0 g) were extracted overnight with a (1:1) mixture of CH_2_Cl_2_/MeOH. After filtration and solvent removal, the alkaloid extract was dissolved in MeOH and NaBH_4_ (500 mg) was slowly added at 0°C. After completion of the reduction (TLC control), water was added and the solution was extracted with CH_2_Cl_2_ (alkaloids were monitored with the Dragendorff reagent). The combined organic layers were dried over MgSO_4_, filtered and concentrated *in vacuo*. The residue was purified by chromatography on silica gel (Eluent CH_2_Cl_2_/MeOH 99/1) to give THA and raubasine as white powders. The former was crystallized from MeOH to give THA as colorless plates suitable for X-ray diffraction.

### 3. Refinement

H3, H15 and H20 atoms bonded to C3, C15 and C20 atoms respectively were located in a difference map and refined isotropically. Other H atoms were positioned geometrically and refined using a riding model.

### Crystal data

**Table:**

C_21_H_24_N_2_O_3_	*F*(000) = 752
*M**_r_* = 352.42	*D*_x_ = 1.250 Mg m^−^^3^
Orthorhombic, *P*2_1_2_1_2_1_	Mo *K*α radiation, λ = 0.71069 Å
Hall symbol: P 2ac 2ab	Cell parameters from 1924 reflections
*a* = 6.719 (1) Å	θ = 0.4–25.4°
*b* = 8.169 (2) Å	µ = 0.08 mm^−^^1^
*c* = 34.120 (5) Å	*T* = 293 K
*V* = 1872.8 (6) Å^3^	Parallelepiped, colourless
*Z* = 4	0.50 × 0.30 × 0.10 mm

### Data collection

**Table:**

Nonius KappaCCD diffractometer	3300 independent reflections
Radiation source: fine-focus sealed tube	2537 reflections with *I* > 2σ(*I*)
Horizonally mounted graphite crystal monochromator	*R*_int_ = 0.000
Detector resolution: 9 pixels mm^-1^	θ_max_ = 25.3°, θ_min_ = 3.5°
CCD scans	*h* = −8→8
Absorption correction: multi-scan (*COLLECT*; Nonius, 2004)	*k* = −9→9
*T*_min_ = 0.982, *T*_max_ = 0.992	*l* = −40→40
3300 measured reflections	

### Refinement

**Table:**

Refinement on *F*^2^	Primary atom site location: structure-invariant direct methods
Least-squares matrix: full	Secondary atom site location: difference Fourier map
*R*[*F*^2^ > 2σ(*F*^2^)] = 0.044	Hydrogen site location: inferred from neighbouring sites
*wR*(*F*^2^) = 0.114	H atoms treated by a mixture of independent and constrained refinement
*S* = 1.01	*w* = 1/[σ^2^(*F*_o_^2^) + (0.0585*P*)^2^ + 0.175*P*] where *P* = (*F*_o_^2^ + 2*F*_c_^2^)/3
3300 reflections	(Δ/σ)_max_ < 0.001
247 parameters	Δρ_max_ = 0.11 e Å^−^^3^
0 restraints	Δρ_min_ = −0.12 e Å^−^^3^

### Special details

**Table:**

Geometry. All s.u.'s (except the s.u. in the dihedral angle between two l.s. planes) are estimated using the full covariance matrix. The cell s.u.'s are taken into account individually in the estimation of s.u.'s in distances, angles and torsion angles; correlations between s.u.'s in cell parameters are only used when they are defined by crystal symmetry. An approximate (isotropic) treatment of cell s.u.'s is used for estimating s.u.'s involving l.s. planes.
Refinement. Refinement of *F*^2^ against ALL reflections. The weighted *R*-factor *wR* and goodness of fit *S* are based on *F*^2^, conventional *R*-factors *R* are based on *F*, with *F* set to zero for negative *F*^2^. The threshold expression of *F*^2^ > 2σ(*F*^2^) is used only for calculating *R*-factors(gt) *etc*. and is not relevant to the choice of reflections for refinement. *R*-factors based on *F*^2^ are statistically about twice as large as those based on *F*, and *R*- factors based on ALL data will be even larger.

### Fractional atomic coordinates and isotropic or equivalent isotropic displacement parameters (Å^2^)

**Table:**

	*x*	*y*	*z*	*U*_iso_*/*U*_eq_	
N1	0.0941 (2)	1.03273 (19)	0.22093 (5)	0.0543 (4)	
H1	0.0106	1.0929	0.2084	0.065*	
C2	0.2643 (3)	0.9635 (2)	0.20539 (6)	0.0498 (4)	
C3	0.3268 (3)	0.9745 (3)	0.16349 (6)	0.0554 (5)	
H3	0.388 (3)	1.082 (3)	0.1594 (6)	0.072 (6)*	
N4	0.4687 (2)	0.8412 (2)	0.15597 (6)	0.0661 (5)	
C5	0.6291 (3)	0.8379 (3)	0.18547 (8)	0.0769 (7)	
H5A	0.6887	0.9457	0.1874	0.092*	
H5B	0.7315	0.7616	0.1772	0.092*	
C6	0.5502 (3)	0.7872 (3)	0.22502 (8)	0.0703 (6)	
H6A	0.5248	0.6704	0.2252	0.084*	
H6B	0.6483	0.8111	0.2451	0.084*	
C7	0.3616 (3)	0.8781 (2)	0.23357 (6)	0.0560 (5)	
C8	0.2480 (3)	0.8919 (2)	0.26876 (6)	0.0561 (5)	
C9	0.2699 (4)	0.8333 (3)	0.30708 (7)	0.0713 (6)	
H9	0.3806	0.7713	0.3139	0.086*	
C10	0.1262 (5)	0.8682 (3)	0.33441 (8)	0.0834 (8)	
H10	0.1396	0.8284	0.3598	0.100*	
C11	−0.0392 (5)	0.9624 (3)	0.32467 (7)	0.0834 (7)	
H11	−0.1357	0.9828	0.3436	0.100*	
C12	−0.0628 (4)	1.0259 (3)	0.28766 (6)	0.0701 (6)	
H12	−0.1717	1.0912	0.2815	0.084*	
C13	0.0803 (3)	0.9893 (2)	0.25995 (6)	0.0542 (5)	
C14	0.1540 (3)	0.9621 (3)	0.13484 (5)	0.0536 (5)	
H14A	0.0620	1.0515	0.1395	0.064*	
H14B	0.0834	0.8602	0.1392	0.064*	
C15	0.2267 (4)	0.9687 (3)	0.09227 (6)	0.0640 (6)	
H15	0.281 (4)	1.076 (3)	0.0888 (7)	0.073 (7)*	
C16	0.0636 (4)	0.9412 (3)	0.06288 (6)	0.0662 (6)	
C17	0.0518 (5)	0.8003 (3)	0.04332 (7)	0.0847 (8)	
H17	−0.0469	0.7924	0.0243	0.102*	
O18	0.1678 (3)	0.6694 (2)	0.04839 (6)	0.1032 (7)	
C19	0.3020 (5)	0.6744 (3)	0.08207 (8)	0.0872 (8)	
H19	0.2254	0.6518	0.1059	0.105*	
C20	0.3918 (4)	0.8443 (3)	0.08520 (8)	0.0797 (7)	
H20	0.465 (4)	0.871 (3)	0.0595 (7)	0.088 (7)*	
C21	0.5520 (4)	0.8600 (4)	0.11650 (8)	0.0887 (8)	
H21A	0.6530	0.7770	0.1122	0.106*	
H21B	0.6151	0.9664	0.1143	0.106*	
C22	0.4475 (7)	0.5356 (4)	0.07534 (11)	0.1426 (15)	
H22A	0.5421	0.5670	0.0556	0.214*	
H22B	0.3765	0.4401	0.0668	0.214*	
H22C	0.5162	0.5117	0.0993	0.214*	
C23	−0.0746 (4)	1.0739 (3)	0.05479 (6)	0.0695 (6)	
O24	−0.2128 (3)	1.0354 (2)	0.02800 (5)	0.0910 (6)	
O25	−0.0672 (3)	1.2070 (2)	0.06996 (5)	0.0945 (6)	
C26	−0.3520 (5)	1.1602 (4)	0.01729 (10)	0.1060 (10)	
H26A	−0.2815	1.2528	0.0069	0.159*	
H26B	−0.4264	1.1930	0.0400	0.159*	
H26C	−0.4414	1.1184	−0.0023	0.159*	

### Atomic displacement parameters (Å^2^)

**Table:**

	*U*^11^	*U*^22^	*U*^33^	*U*^12^	*U*^13^	*U*^23^
N1	0.0552 (9)	0.0540 (8)	0.0537 (9)	0.0087 (8)	−0.0107 (8)	−0.0008 (8)
C2	0.0463 (10)	0.0450 (9)	0.0581 (11)	0.0012 (9)	−0.0105 (9)	−0.0051 (8)
C3	0.0481 (11)	0.0531 (11)	0.0651 (13)	−0.0025 (10)	−0.0025 (9)	−0.0021 (10)
N4	0.0456 (9)	0.0757 (12)	0.0770 (13)	0.0066 (9)	0.0038 (9)	−0.0051 (9)
C5	0.0452 (12)	0.0821 (16)	0.103 (2)	0.0048 (12)	−0.0055 (13)	−0.0034 (15)
C6	0.0513 (12)	0.0617 (12)	0.0980 (18)	0.0078 (11)	−0.0201 (13)	−0.0022 (12)
C7	0.0530 (11)	0.0462 (10)	0.0687 (14)	0.0017 (9)	−0.0153 (10)	−0.0056 (9)
C8	0.0641 (12)	0.0417 (9)	0.0625 (13)	−0.0021 (10)	−0.0205 (11)	−0.0017 (9)
C9	0.0917 (17)	0.0530 (12)	0.0691 (15)	−0.0038 (13)	−0.0249 (14)	0.0064 (11)
C10	0.125 (2)	0.0657 (14)	0.0599 (15)	−0.0183 (17)	−0.0172 (16)	0.0068 (11)
C11	0.115 (2)	0.0756 (14)	0.0595 (14)	−0.0120 (18)	0.0064 (14)	−0.0098 (12)
C12	0.0783 (15)	0.0660 (12)	0.0661 (14)	0.0020 (13)	−0.0016 (12)	−0.0122 (11)
C13	0.0597 (11)	0.0488 (10)	0.0540 (11)	0.0013 (9)	−0.0095 (9)	−0.0051 (8)
C14	0.0508 (11)	0.0587 (11)	0.0512 (11)	0.0008 (10)	0.0007 (9)	−0.0045 (9)
C15	0.0718 (15)	0.0622 (12)	0.0579 (12)	−0.0037 (13)	0.0122 (11)	0.0005 (10)
C16	0.0851 (16)	0.0686 (14)	0.0451 (11)	−0.0008 (13)	0.0087 (11)	0.0000 (10)
C17	0.116 (2)	0.0844 (17)	0.0538 (14)	0.0096 (18)	−0.0026 (14)	−0.0102 (12)
O18	0.1444 (18)	0.0837 (12)	0.0815 (13)	0.0226 (13)	−0.0117 (13)	−0.0251 (10)
C19	0.116 (2)	0.0745 (16)	0.0709 (16)	0.0229 (16)	0.0094 (16)	−0.0093 (12)
C20	0.0769 (16)	0.0944 (18)	0.0676 (15)	0.0116 (15)	0.0276 (14)	−0.0005 (13)
C21	0.0621 (15)	0.111 (2)	0.0934 (19)	0.0084 (16)	0.0258 (15)	−0.0032 (16)
C22	0.191 (4)	0.117 (2)	0.120 (3)	0.072 (3)	−0.003 (3)	−0.029 (2)
C23	0.0886 (17)	0.0782 (16)	0.0418 (11)	0.0033 (14)	0.0077 (12)	0.0046 (10)
O24	0.1015 (13)	0.1035 (13)	0.0679 (10)	0.0172 (12)	−0.0157 (9)	−0.0089 (9)
O25	0.1331 (17)	0.0745 (10)	0.0759 (12)	0.0125 (12)	−0.0110 (11)	−0.0040 (9)
C26	0.102 (2)	0.122 (2)	0.094 (2)	0.027 (2)	−0.0055 (17)	0.0224 (17)

### Geometric parameters (Å, º)

**Table:**

N1—C2	1.382 (3)	C14—H14A	0.9700
N1—C13	1.381 (2)	C14—H14B	0.9700
N1—H1	0.8600	C15—C16	1.502 (3)
C2—C7	1.356 (3)	C15—C20	1.524 (3)
C2—C3	1.492 (3)	C15—H15	0.96 (2)
C3—N4	1.470 (3)	C16—C17	1.333 (3)
C3—C14	1.521 (3)	C16—C23	1.454 (3)
C3—H3	0.98 (2)	C17—O18	1.334 (3)
N4—C21	1.466 (3)	C17—H17	0.9300
N4—C5	1.475 (3)	O18—C19	1.461 (3)
C5—C6	1.508 (3)	O18—O24^i^	3.199 (2)
C5—H5A	0.9700	C19—C22	1.515 (4)
C5—H5B	0.9700	C19—C20	1.517 (4)
C6—C7	1.497 (3)	C19—H19	0.9800
C6—H6A	0.9700	C20—C21	1.522 (4)
C6—H6B	0.9700	C20—H20	1.03 (3)
C7—C8	1.427 (3)	C21—H21A	0.9700
C8—C9	1.400 (3)	C21—H21B	0.9700
C8—C13	1.412 (3)	C22—H22A	0.9600
C9—C10	1.372 (4)	C22—H22B	0.9600
C9—H9	0.9300	C22—H22C	0.9600
C10—C11	1.392 (4)	C23—O25	1.205 (3)
C10—H10	0.9300	C23—O24	1.340 (3)
C11—C12	1.374 (3)	O24—C26	1.430 (3)
C11—H11	0.9300	C26—H26A	0.9600
C12—C13	1.381 (3)	C26—H26B	0.9600
C12—H12	0.9300	C26—H26C	0.9600
C14—C15	1.533 (3)		
			
C2—N1—C13	108.68 (16)	C15—C14—H14B	109.4
C2—N1—H1	125.7	H14A—C14—H14B	108.0
C13—N1—H1	125.7	C16—C15—C20	109.01 (19)
C7—C2—N1	109.74 (19)	C16—C15—C14	113.25 (19)
C7—C2—C3	125.07 (18)	C20—C15—C14	111.00 (19)
N1—C2—C3	125.14 (17)	C16—C15—H15	109.3 (14)
N4—C3—C2	107.76 (17)	C20—C15—H15	108.5 (14)
N4—C3—C14	109.48 (16)	C14—C15—H15	105.7 (14)
C2—C3—C14	113.41 (16)	C17—C16—C23	120.7 (2)
N4—C3—H3	111.4 (13)	C17—C16—C15	120.5 (2)
C2—C3—H3	107.9 (13)	C23—C16—C15	118.75 (19)
C14—C3—H3	106.9 (13)	C16—C17—O18	126.3 (3)
C21—N4—C3	109.28 (18)	C16—C17—H17	116.8
C21—N4—C5	110.49 (19)	O18—C17—H17	116.8
C3—N4—C5	111.60 (17)	C17—O18—C19	116.12 (18)
N4—C5—C6	111.05 (18)	C17—O18—O24^i^	117.37 (15)
N4—C5—H5A	109.4	C19—O18—O24^i^	120.05 (15)
C6—C5—H5A	109.4	O18—C19—C22	105.0 (2)
N4—C5—H5B	109.4	O18—C19—C20	109.0 (2)
C6—C5—H5B	109.4	C22—C19—C20	116.0 (3)
H5A—C5—H5B	108.0	O18—C19—H19	108.9
C7—C6—C5	109.63 (18)	C22—C19—H19	108.9
C7—C6—H6A	109.7	C20—C19—H19	108.9
C5—C6—H6A	109.7	C19—C20—C21	114.0 (2)
C7—C6—H6B	109.7	C19—C20—C15	109.4 (2)
C5—C6—H6B	109.7	C21—C20—C15	110.4 (2)
H6A—C6—H6B	108.2	C19—C20—H20	108.9 (14)
C2—C7—C8	107.32 (17)	C21—C20—H20	104.1 (14)
C2—C7—C6	121.7 (2)	C15—C20—H20	109.9 (14)
C8—C7—C6	130.99 (19)	N4—C21—C20	111.5 (2)
C9—C8—C13	118.4 (2)	N4—C21—H21A	109.3
C9—C8—C7	134.6 (2)	C20—C21—H21A	109.3
C13—C8—C7	107.01 (17)	N4—C21—H21B	109.3
C10—C9—C8	119.3 (2)	C20—C21—H21B	109.3
C10—C9—H9	120.3	H21A—C21—H21B	108.0
C8—C9—H9	120.3	C19—C22—H22A	109.5
C9—C10—C11	120.9 (2)	C19—C22—H22B	109.5
C9—C10—H10	119.5	H22A—C22—H22B	109.5
C11—C10—H10	119.5	C19—C22—H22C	109.5
C12—C11—C10	121.3 (2)	H22A—C22—H22C	109.5
C12—C11—H11	119.3	H22B—C22—H22C	109.5
C10—C11—H11	119.3	O25—C23—O24	122.2 (2)
C11—C12—C13	117.8 (2)	O25—C23—C16	124.4 (2)
C11—C12—H12	121.1	O24—C23—C16	113.4 (2)
C13—C12—H12	121.1	C23—O24—C26	117.4 (2)
C12—C13—N1	130.62 (19)	O24—C26—H26A	109.5
C12—C13—C8	122.13 (19)	O24—C26—H26B	109.5
N1—C13—C8	107.25 (18)	H26A—C26—H26B	109.5
C3—C14—C15	111.33 (17)	O24—C26—H26C	109.5
C3—C14—H14A	109.4	H26A—C26—H26C	109.5
C15—C14—H14A	109.4	H26B—C26—H26C	109.5
C3—C14—H14B	109.4		
			
C13—N1—C2—C7	−0.7 (2)	C7—C8—C13—N1	−0.3 (2)
C13—N1—C2—C3	176.71 (18)	N4—C3—C14—C15	−57.9 (2)
C7—C2—C3—N4	17.0 (3)	C2—C3—C14—C15	−178.22 (18)
N1—C2—C3—N4	−159.97 (17)	C3—C14—C15—C16	174.62 (19)
C7—C2—C3—C14	138.4 (2)	C3—C14—C15—C20	51.6 (3)
N1—C2—C3—C14	−38.6 (3)	C20—C15—C16—C17	17.6 (3)
C2—C3—N4—C21	−173.13 (18)	C14—C15—C16—C17	−106.5 (3)
C14—C3—N4—C21	63.1 (2)	C20—C15—C16—C23	−159.33 (19)
C2—C3—N4—C5	−50.6 (2)	C14—C15—C16—C23	76.6 (3)
C14—C3—N4—C5	−174.38 (18)	C23—C16—C17—O18	−179.2 (2)
C21—N4—C5—C6	−169.4 (2)	C15—C16—C17—O18	4.0 (4)
C3—N4—C5—C6	68.8 (2)	C16—C17—O18—C19	8.7 (4)
N4—C5—C6—C7	−45.6 (2)	C16—C17—O18—O24^i^	−143.1 (2)
N1—C2—C7—C8	0.5 (2)	C17—O18—C19—C22	−166.5 (3)
C3—C2—C7—C8	−176.90 (18)	O24^i^—O18—C19—C22	−15.5 (3)
N1—C2—C7—C6	178.97 (16)	C17—O18—C19—C20	−41.6 (3)
C3—C2—C7—C6	1.6 (3)	O24^i^—O18—C19—C20	109.46 (19)
C5—C6—C7—C2	12.5 (3)	O18—C19—C20—C21	−173.59 (19)
C5—C6—C7—C8	−169.4 (2)	C22—C19—C20—C21	−55.4 (3)
C2—C7—C8—C9	−179.6 (2)	O18—C19—C20—C15	62.3 (3)
C6—C7—C8—C9	2.1 (4)	C22—C19—C20—C15	−179.5 (2)
C2—C7—C8—C13	−0.1 (2)	C16—C15—C20—C19	−49.3 (3)
C6—C7—C8—C13	−178.41 (19)	C14—C15—C20—C19	76.1 (3)
C13—C8—C9—C10	1.8 (3)	C16—C15—C20—C21	−175.5 (2)
C7—C8—C9—C10	−178.8 (2)	C14—C15—C20—C21	−50.1 (3)
C8—C9—C10—C11	−0.7 (3)	C3—N4—C21—C20	−63.3 (3)
C9—C10—C11—C12	−1.1 (4)	C5—N4—C21—C20	173.5 (2)
C10—C11—C12—C13	1.8 (3)	C19—C20—C21—N4	−67.0 (3)
C11—C12—C13—N1	178.8 (2)	C15—C20—C21—N4	56.6 (3)
C11—C12—C13—C8	−0.7 (3)	C17—C16—C23—O25	−176.4 (3)
C2—N1—C13—C12	−179.0 (2)	C15—C16—C23—O25	0.5 (3)
C2—N1—C13—C8	0.6 (2)	C17—C16—C23—O24	2.8 (3)
C9—C8—C13—C12	−1.0 (3)	C15—C16—C23—O24	179.7 (2)
C7—C8—C13—C12	179.33 (19)	O25—C23—O24—C26	1.0 (4)
C9—C8—C13—N1	179.34 (18)	C16—C23—O24—C26	−178.2 (2)

Symmetry code: (i) *x*+1/2, −*y*+3/2, −*z*.

### Hydrogen-bond geometry (Å, º)

Cg1 and Cg2 are the centroids of the C8–C13 and C2/C7/C8/C13/N1 rings, respectively.

**Table:**

*D*—H···*A*	*D*—H	H···*A*	*D*···*A*	*D*—H···*A*
N1—H1···*Cg*1^ii^	0.86	2.85	3.550 (2)	139
C6—H6*A*···*Cg*2^iii^	0.97	2.8	3.429 (3)	121

Symmetry codes: (ii) −*x*, *y*+1/2, −*z*+1/2; (iii) −*x*+1, *y*−1/2, −*z*+1/2.
